# Posture Allocation Revisited: Breaking the Sedentary Threshold of Energy Expenditure for Obesity Management

**DOI:** 10.3389/fphys.2017.00420

**Published:** 2017-06-22

**Authors:** Jennifer L. Miles-Chan, Abdul G. Dulloo

**Affiliations:** Department of Medicine Physiology, University of FribourgFribourg, Switzerland

**Keywords:** energy expenditure, posture, obesity, spontaneous physical activity, thermogenesis

## Abstract

There is increasing recognition that low-intensity physical activities of daily life play an important role in achieving energy balance and that their societal erosion through substitution with sedentary (mostly sitting) behaviors, whether occupational or for leisure, impact importantly on the obesity epidemic. This has generated considerable interest for better monitoring, characterizing, and promoting countermeasures to sedentariness through a plethora of low-level physical activities (e.g., active workstations, standing desks, sitting breaks), amid the contention that altering posture allocation (lying, sitting, standing) can modify energy expenditure to impact upon body weight regulation and health. In addressing this contention, this paper first revisits the past and more recent literature on postural energetics, with particular emphasis on potential determinants of the large inter-individual variability in the energy cost of standing and the impact of posture on fat oxidation. It subsequently analyses the available data pertaining to various strategies by which posture allocations, coupled with light physical activity, may increase energy expenditure beyond the sedentary threshold, and their relevance as potential targets for obesity management.

Evolutionary scientists still are not sure why our ancestors became bipedal, but along with the evolution of the major traits and behaviors that define humans (such as large brains, language, art, technology), walking upright - and the performance of a plethora of activities while maintaining standing posture—is a most fundamental human characteristic (Wayman, 2012). Yet, Modern Man (and Woman) is sedentary for much larger proportions of the day than ever before (Ng and Popkin, 2012). Indeed, a modern lifestyle involves a large variety of seated activities, whether they be occupational or for leisure. The rise in the prevalence of such activities has led to the notion of a major shift in posture allocation from standing in favor of sitting on a population basis. With this belief has come a myriad of correlative analyses showing a positive relationship between sitting time and cardiometabolic disease risk (Henson et al., 2016; Young et al., 2016; Tigbe et al., 2017). In addition, studies have now shown that obese individuals spend significantly more time sitting and less time standing than their lean counterparts (Levine et al., 2005; Johannsen et al., 2008). This therefore begs the question as to whether or not modifying posture allocation could sufficiently alter energy expenditure (EE) in order to impact body weight regulation over time; an idea that requires us to revisit the literature concerning postural energetics.

## Historical interest in posture allocation

Whilst, the interest in posture allocation as a potential target in obesity prevention has increased over recent years, interest in quantifying its energetic cost originated in an entirely different scientific and social context.

During the first half of the twentieth century there was considerable attention on improving guidelines of energy requirements at the individual and population level; with such information required to provide aid and assistance to developing and war-torn countries as well as to optimize military performance. A major hurdle in estimating energy requirements was the need to establish a database of the energy cost of common, standardized physical activities. The breakthrough came in the 1940s with the development of the Kofranyi-Michaelis or Max Planck respirometer (Passmore and Durnin, 1955). Despite being comparatively heavy compared to modern devices, this respirometer allowed researchers for the first time to measure EE by indirect calorimetry during a host of daily-life activities, in the field, and in very diverse populations (for example: Passmore et al., 1952; Passmore and Durnin, 1955).

It is noticeable from these early studies that considerable emphasis was put on variability in the energy cost of standardized low-level physical activities both between and within individuals—an important aspect of human energetics which has been largely overlooked in more recent studies. For example, the classic studies of Passmore et al. (1952) and Edholm et al. (1955) both reported large inter-individual variability in the energy cost for performing the same activity, with EE during standing compared to sitting increasing by anything from ~0% to >30% in individuals from relatively homogenous study groups. In addition to this inter-individual variability, Miller (1982) reported intra-individual variability in the energy cost of sitting and standing in six individuals to range from 5 to 13% and 4 to 7%, respectively.

**Figure 1:**
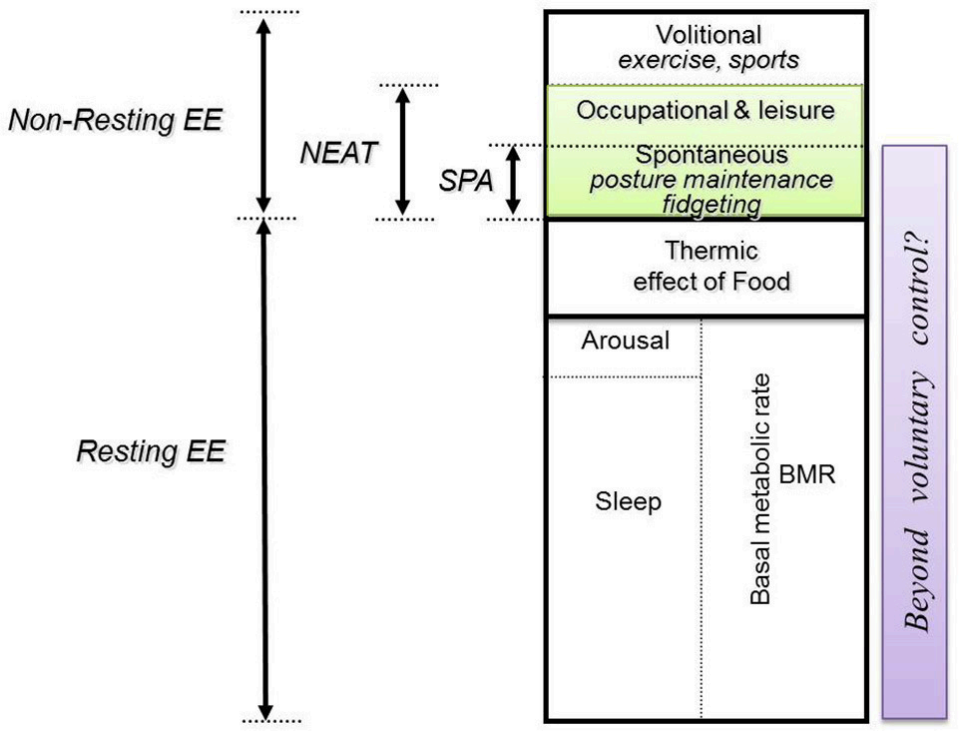
Compartments of daily energy expenditure. Daily energy expenditure (EE) can be divided into resting and non-resting EE. Non-resting EE can further be divided into (i) volitional EE related to structured physical activities, such as sports and exercise, which are usually of moderate-to-vigorous intensity; and (ii) non-exercise activities (NEAT). These non-exercise activities include both those under voluntary (conscious) control associated with occupation and leisure, and those that are involuntary (subconscious) in nature. This sub-compartment of spontaneous physical activity (SPA) includes low-level physical activities such as postural maintenance and fidgeting. Adapted from Dulloo et al. (2012).

It was not until the demonstration by Zurlo et al. (1992) of an inverse correlation between spontaneous physical activity (SPA) and body weight gain in Pima Indians that research interest in low-level physical activities and sedentary behaviors in the context of obesity development really began; SPA being a term that encapsulates posture maintenance, fidgeting and other essentially subconscious low-level movement (Dulloo et al., 2012). However, the watershed moment occurred at the turn of this century, with the observation by Levine et al. (1999) of an increase in non-exercise activity thermogenesis (NEAT) in individuals showing a relative resistance to fat gain during overfeeding; NEAT being estimated by subtraction of basal and postprandial EE from total daily EE. Whilst posture allocation is just one component of SPA and NEAT (Figure 1), two subsequent studies (Levine et al., 2005; Johannsen et al., 2008), each involving 10 lean and 10 obese individuals, have provided evidence of a difference in posture allocation between these two population groups—therefore highlighting a new potential target for obesity treatment and prevention.

## Energy cost of posture maintenance

As a result of these two observational studies, it has been suggested that if obese individuals were to match the posture allocation of lean individuals—i.e., by re-allocating 2–2.5 h of sitting time to standing per day—then daily EE would be increased by ~300–350 kcal or ~10–20% (Levine et al., 2005; Johannsen et al., 2008); potentially resulting in a weight loss of ~15 kg over a year (Levine et al., 2005). These calculations are based on the following three key assumptions:

1. That standing is not a sedentary behavior, and as such its energetic cost is more than 1.5 times the energy cost of sitting at rest (i.e., >1.5 METs);
2. That the energy cost of standing is constant across the entire standing period regardless of duration; and,
3. That the energy cost of standing is the same or similar between individuals.

However, our analysis of the available literature reveals a number of challenges to these assumptions; these are elaborated below.

### Energy cost of steady-state standing posture maintenance

Since 1952, there have been just over 30 studies presenting measurements of both the energetic cost of standing and sitting, comprising of >60 experimental groups (Table 1; Passmore et al., 1952; Donald and Davidson, 1954; Edholm et al., 1955; Garry et al., 1955; Durnin and Passmore, 1967; Banerjee et al., 1971; Viteri et al., 1971; Banerjee and Saha, 1972; Norgan et al., 1974; Malhotra et al., 1976; de Guzman et al., 1978, 1979, 1984; Bandyopadhyay and Chattopadhyay, 1980; Bleiberg et al., 1980; Brun et al., 1981; Geissler et al., 1981; Geissler and Aldouri, 1985; Lawrence et al., 1985; Cole and Ogbe, 1987; Edmundson and Edmundson, 1988; Strickland and Ulijaszek, 1990; Li and Yan, 1991; Katzmarzyk et al., 1996; Levine et al., 2000; Sujatha et al., 2000; Kanade et al., 2001; Levine and Miller, 2007; McAlpine et al., 2007; Beers et al., 2008; Rao et al., 2008; Speck and Schmitz, 2011; Reiff et al., 2012; Steeves et al., 2012; Whybrow et al., 2013; Buckley et al., 2014; Creasy et al., 2016; Fountaine et al., 2016; Judice et al., 2016; Gibbs et al., 2017).

**Table 1:** Table of existing studies published from 1952 to 2017, specifying the values of energy expenditure during sitting vs. standing.

**Study**	**Ethnicity (if stated)**	***n* (gender)**	**Mean EE**		**ΔEE from sitting (%)**	**Length of measurement (if stated)**
			**Sitting**	**Standing**		
Bandyopadhyay and Chattopadhyay, 1980	Indian	11♂	1.01 kcal/min	1.07 kcal/min	5.9	–
Banerjee et al., 1971	Asian	37♀	0.90 kcal/min	1.02 kcal/min	13.3	–
Banerjee and Saha, 1972	–	10♂	1.03 kcal/min	1.39 kcal/min	35	5 min
		10♀	0.68 kcal/min	0.89 kcal/min	30.9	~10 min
Beers et al., 2008	–	12♂	80 kcal/h^*^	86 kcal/h^*^	7.5	Last 15 min of 20 min of activity
		12♀	55 kcal/h^*^	57 kcal/h^*^	3.6	Last 15 min of 20 min of activity
Bleiberg et al., 1980	African	27♀	1.29 kcal/min	1.35 kcal/min	4.7	~10 min
Brun et al., 1981	African	29–33♂	1.38 kcal/min	1.44 kcal/min	4.3	~10 min
Buckley et al., 2014	–	8♀, 2♂	1.49 kcal/min	2.32 kcal/min	56	210 mins!
Cole and Ogbe, 1987	African	7–10♂	5.55 kJ/min	11.51 kJ/min	107.4	10–15 min
Creasy et al., 2016	–	7♂, 11♀	1.31 kcal/min	1.43 kcal/min	9.2	15 min
de Guzman et al., 1978	Filipino	10♂	0.026 kcal/kg/min	0.029 kcal/kg/min	11.5	8–10 min
		10♀	0.026 kcal/kg/min	0.027 kcal/kg/min	3.8	
de Guzman et al., 1979	Filipino	25♀	0.022 kcal/kg/min	0.024 kcal/kg/min	9.1	10 min
		14♂	0.024 kcal/kg/min	0.024 kcal/kg/min	0	
de Guzman et al., 1984	Filipino	10♂	0.027 kcal/kg/min	0.027 kcal/kg/min	0	8–10 min
		10♀	0.027 kcal/kg/min	0.029 kcal/kg/min	7.4	
Donald and Davidson, 1954	–	13♂	0.25 L/min	0.27 L/min	8	–
Durnin and Passmore, 1967	–	♂	1.28 kcal/kg weight/h	1.61 kcal/kg weight/h	25.8	–
		♀	1.25 kcal/kg weight/h	1.49 kcal/kg weight/h	19.2	
Edholm et al., 1955	–	12♂	1.60 kcal/min	1.82 kcal/min	13.8	–
Edmundson and Edmundson, 1988	Indian	20–24♂	0.026 kcal/kg/min	0.03 kcal/kg/min	15.4	
Fountaine et al., 2016	–	10♂, 8♀	1.69 kcal/min	1.86 kcal/min	10	20 min
Garry et al., 1955	Scottish	10♂	1.6 kcal/min	1.8 kcal/min	12.5	
Gibbs et al., 2017	Mixed	9♂, 9♀	1.18 kcal/min	1.32 kcal/min	11.5	60 min
Geissler et al., 1981	Iranian	32♀/18♀	1.14 kcal/min	1.24 kcal/min	8.8	10–15 min
Geissler and Aldouri, 1985	European	15♂	1.42 kcal/min	1.59 kcal/min	12	10 min
	Asian	15♂	1.31 kcal/min	1.42 kcal/min	8.4	
	African	15♂	1.29 kcal/min	1.49 kcal/min	15.5	
Judice et al., 2016	–	25♂	1.14 kcal/min	1.23 kcal/min	~8	10 min
		25♀	0.88 kcal/min	0.92 kcal/min	~6.0	
Kanade et al., 2001	Indian	24♂	5.00 kJ/min	5.74 kJ/min	14.8	
		40♀	4.03 kJ/min	4.35 kJ/min	7.9	
Katzmarzyk et al., 1996	Siberia	30♂	6.67 kJ/min	7.09 kJ/min	6.3	3 min
		14♀	4.97 kJ/min	5.35 kJ/min	7.6	
	Highland Ecuador	17♂	6.74 kJ/min	6.84 kJ/min	1.5	
		12♀	5.60 kJ/min	4.94 kJ/min	−11.8	
	Coastal Ecuador	5♂	7.42 kJ/min	7.00 kJ/min	–5.7	
		5♀	6.22 kJ/min	5.64 kJ/min	–9.3	
Lawrence et al., 1985	African	113♀	1.25 kcal/min	1.26 kcal/min	0.8	Last 5 mins of 8 min activity
Levine and Miller, 2007	–	14♀, 1♂	1.20 kcal/min	1.37 kcal/min	14.2	20 min
Levine et al., 2000	White	7♂, 17♀	5.6 kJ/min	6.1 kJ/min	8.9	20 min
Li and Yan, 1991	Chinese	319♂	0.839 kcal/m^2^/min	0.886 kcal/m^2^/min	5.6	Unknown
		287♀	0.818 kcal/m^2^/min	0.846 kcal/m^2^/min	3.4	
Malhotra et al., 1976	Indian	24♂	4.85 kJ/min	5.31 kJ/min	9.5	Unknown
McAlpine et al., 2007	–	11♂, 8♀	1.47 kcal/min	1.62 kcal/min	10.2	20 min
Norgan et al., 1974	New Guinean (Kaul)	40–41♂	1.23 kcal/min	1.32 kcal/min	7.3	Unknown
		41♀	1.08 kcal/min	1.19 kcal/min	10.2	
	New Guinean (Lufa)	32–34♂	1.36 kcal/min	1.47 kcal/min	8.1	
		29–30♀	1.21 kcal/min	1.29 kcal/min	6.6	
Passmore et al., 1952	–	5 ♂	1.82 kcal/min	2.02 kcal/min	10.9	6–15 min
Rao et al., 2008	Indian	4♀/3♀	3.66 kJ/min	4.10 kJ/min	12	6 min
Reiff et al., 2012	–	10♂, 10♀	1.02 kcal/min	1.36 kcal/min	33.3	last 30 min of 45 min activity
Speck and Schmitz, 2011	Indian	5♂, 8♀	1.26 kcal/min	1.287 kcal/min	2.1	6 min
Steeves et al., 2012	–	11♂, 12♀	86 kcal/h^*^	93 kcal/h^*^	8.1	5 min
Strickland and Ulijaszek, 1990	Gurkha	11♂	6.3 kJ/min	6.7 kJ/min	6.3	10 min
	British	11♂	5.9 kJ/min	6.8 kJ/min	15.3	
Sujatha et al., 2000	Indian	98♀	3.15 kJ/min	3.430 kJ/min	8.8	6 min
Viteri et al., 1971	Central American	18–19♂	1.21 kcal/min	1.28 kcal/min	5.8	Last 10 min of 15–20 min activity
Whybrow et al., 2013	–	7♂, 7♀	6.1 kJ/min	6.9 kJ/min	13.1	5 min

*Information obtained from original reference or compilations of Passmore and Durnin (1955) and Vaz et al. (2005)*.

* *Estimated from graphic*.

**Figure 2:**
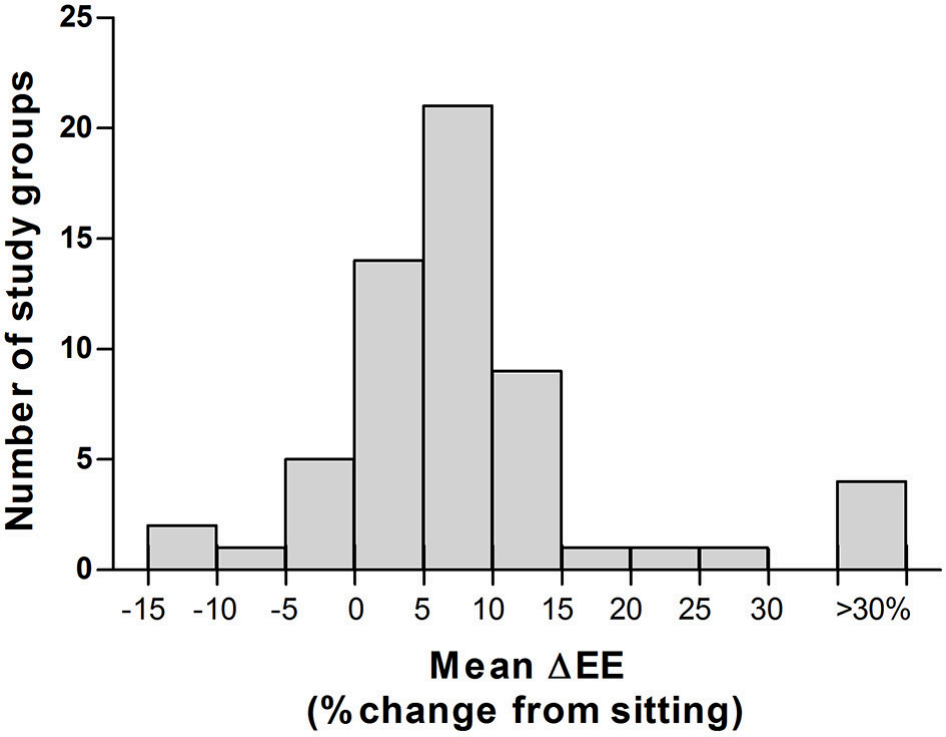
Inter-study variability in the energy cost of standing vs. sitting. Histogram of all energy cost of standing vs sitting reported by all studies published 1952–2017 (*n* = 32 studies, 59 study groups). Mean ± SEM: 11.6 ± 2.1%; Median: 8.4%; Range −11.8% to +107.4%. Please refer to Table 1 for further details of individual studies.

By comparing these values of standing relative to sitting (Figure 2), we can observe considerable variability amongst these studies, with the energy cost of standing ranging from a 10% decrease in EE during standing relative to sitting (measured in females of two subsistence-level populations in Ecuador; Katzmarzyk et al., 1996) to increases in EE of >30% above sitting values (with one study observing a mean increase of >100%; Cole and Ogbe, 1987); with an overall mean increase in EE during standing posture maintenance of 11.6%, and a median increase of 8.6%, above sitting EE. It is important to note that these studies differed considerably in terms of methodology, their level of standardization, presentation of results (i.e., integrated mean over entire standing period vs. average of last 5 min) and their definition of standing itself (i.e., with or without fidgeting, length of standing period), thus making direct comparison between these studies difficult. However, regardless of these inconsistencies, it appears that the true energy cost of steady-state standing posture maintenance is considerably lower than the commonly described sedentary threshold of 1.5 METs (Sedentary Behaviour Research Network, 2012).

### Time-course of energy cost of standing posture maintenance

**Figure 3:**
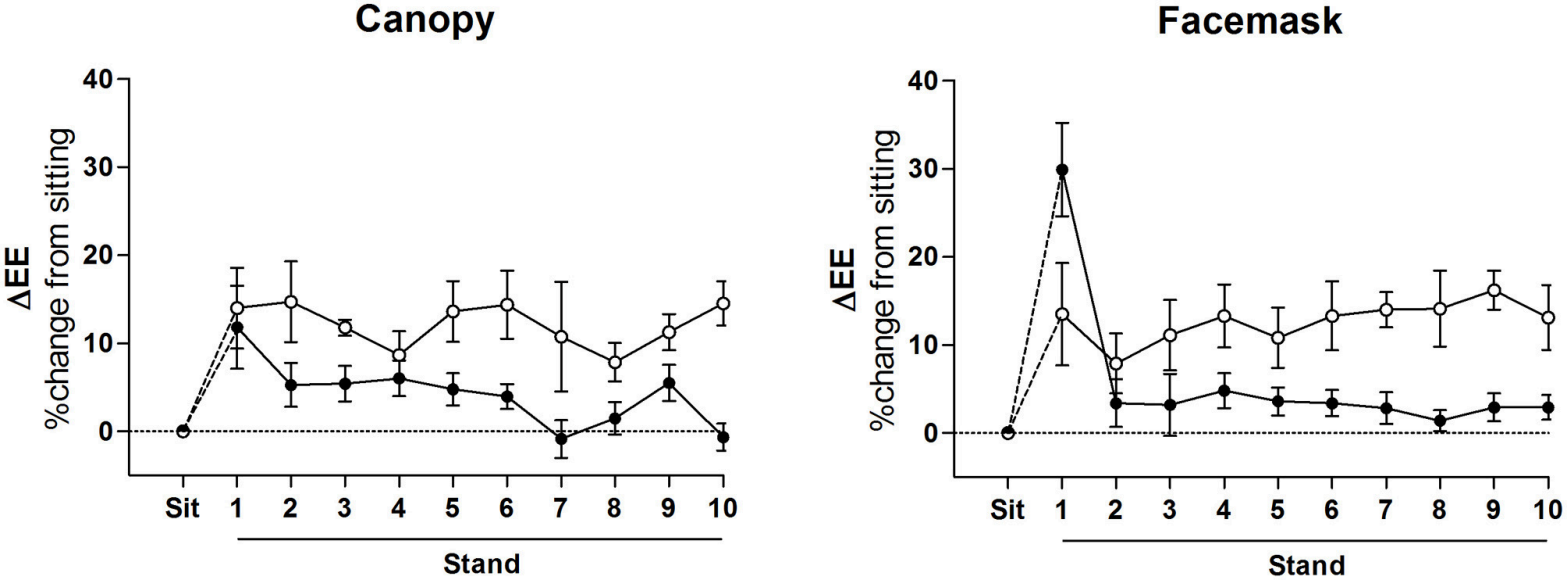
Time course of energy cost of standing posture maintenance. Change in energy expenditure (EE) measured during 10 min of steady-state standing (i.e., after postural transition) as a percentage of resting, sitting EE. Mean ± SEM. **Left panel**: measurements obtained using posture-adapted ventilated canopy indirect calorimetry (Deltatrac II, Datex-Ohmeda, Instrumentarium Corp, Helsinki, Finland) (Miles-Chan et al., 2013); **Right panel**: measurements obtained using facemask indirect calorimetry (Cosmed Quark, Cosmed srl, Rome, Italy) (Miles-Chan et al., 2017); Closed circles: represent “Energy-savers,” i.e., those who showed little or no change in EE (a rise in EE of <5%) during 10 min standing period relative to sitting, and also those who increased EE (a rise in EE of >5%) during first 5 min of the 10 min standing period relative to sitting but subsequently decreased EE (by >30% of the rise) during the second 5 min of this standing period; *n* = 18 (**left panel**) and 29 (**right panel**); *Open circles:* represent “Energy-spenders,” i.e., those who increased EE (a rise in EE of >5%) during first 5 min of the 10 min standing period relative to sitting, and maintained an elevated EE throughout the entire 10 min standing period (drop in EE during second 5 min <30% of the rise in EE during first 5 of standing period); *n* = 4 (**left panel**) and 7 (**right panel**).

Investigations of the energy cost of standing posture maintenance almost exclusively present the EE during standing (and therefore the calculation of its energy cost) as an integrated mean across the entire standing period, regardless of its duration. However, there seems to be little evidence to support the notion that EE is indeed constant during standing. In fact studies conducted in our laboratory using minute-by-minute EE monitoring have shown that the majority of individuals demonstrate an initial increase in EE (most likely due to the postural transition) and then rather quickly (within 5 min) decrease their EE back to sitting values (Miles-Chan et al., 2013, 2017; Monnard and Miles-Chan, 2017; Figure 3). The rise in EE during postural transitioning is expected given the large amount of muscular contraction required to move the body weight from, for example, a sitting to standing position; but it is perhaps inclusion of this transitional period of EE, rather than consideration of only the steady-state period of posture maintenance, that has led to some of the large discrepancies in calculated energy costs.

The exact mechanisms by which the majority of individuals are able to maintain a standing posture at the same energetic cost as sitting remain to be elucidated, although it appears somewhat analogous to the adaptation in energy cost observed during other physical activities. For example, a large volume of research now supports the notion that locomotion is quickly and precisely optimized in order to minimize its energetic cost. Such optimization may occur in response factors like pregnancy (Poppitt et al., 1993), load-carrying (Maloiy et al., 1986; Jones et al., 1987; Lloyd et al., 2010), or exogenous gait disturbance (Koller et al., 2015; Selinger et al., 2015), and is not unique to humans—with locomotive optimization demonstrated across a large number of species (Tucker, 1970; Alexander, 1989). Also, importantly when considering time-course of relatively short physical activities such as standing maintenance, recent studies involving the perturbation of human gait have shown that adaptations that minimize energetic cost of locomotion occur within minutes (Selinger et al., 2015); i.e., within the timescale over which standing is usually performed.

### Variability in energy cost of standing posture maintenance

**Figure 4:**
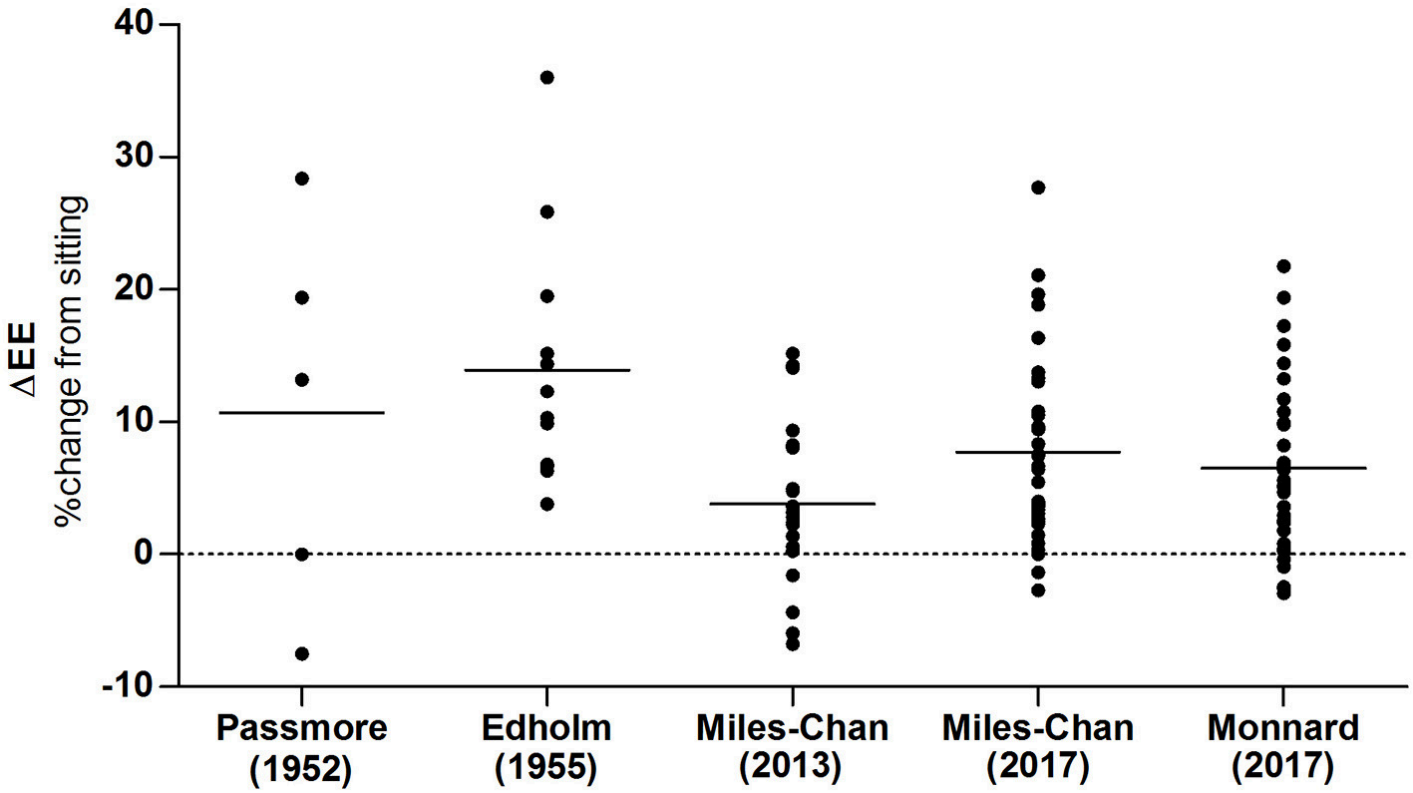
Inter-individual (intra-study) variability in the energy cost of standing vs. sitting. Scatter plot of variability in the energy cost of standing vs. sitting between individual subjects in the studies of Edholm et al. (1955), Passmore et al. (1952), Miles-Chan et al. (2013), Miles-Chan et al. (2017), and Monnard and Miles-Chan (2017). Each point represents an individual study participant; horizontal lines indicate median and interquartile range.

As discussed earlier, there is considerable variability in the energy cost of posture maintenance in healthy individuals. Whilst a certain amount of variability may be accounted for by differences in standardization and methodology, large levels of within-study variability (i.e., amongst individuals measured under identical experimental conditions) strongly suggests a large degree of true biological variability. Indeed the inter-individual variability shown in the early studies of Edholm et al. (1955) and Passmore et al. (1952), is almost identical to that which we have recently observed in our laboratory using contemporary equipment (Miles-Chan et al., 2013, 2017; Monnard and Miles-Chan, 2017)—i.e., ranging from individuals who showed no increase in EE during steady-state standing relative to sitting (“energy savers”) to those who showed sustained increases in EE of 25–35% (“energy spenders;” Figure 4). This is in sharp contrast to a relatively low intra-individual coefficient of variation in the energy cost of standing—reported by Miller to range from 4 to 7% (Miller, 1982), and the intra-individual coefficient of variation in EE during standing within our own laboratory to range from 0 to 7% (Miles-Chan et al., 2017). Nevertheless, using standardized experimental conditions, we have yet to observe any difference in terms of sex (Miles-Chan et al., 2013, 2017) or ethnic group (Monnard and Miles-Chan, 2017) between these two EE phenotypes. Furthermore, given that during standing posture maintenance individuals appear to differ in terms of the degree and pattern of weight-shifting behavior (i.e., the redistribution of body-weight from one foot to the other), we have recently investigated if an overt difference in terms of spontaneous weight-shifting behavior could be detected between these two EE phenotypes (Miles-Chan et al., 2017). However, no such difference was apparent amongst the healthy young adults who participated in the study. It therefore remains unclear as to whether or not this apparent adaptive failure resides in a physiological difference between “energy spenders” and “energy savers,” is related to psychological factors (for example, a strong preference for one posture over the other), or a combination of the two.

Moreover, given earlier demonstrations that the energy cost of physical activities such as walking may vary by 46% depending on energy intake (Apfelbaum et al., 1971), further investigations are warranted to assess the energy cost of standing posture maintenance in the postprandial phase, particularly given that the majority of the day is spent in the absorptive state. But perhaps most importantly, given the postulation that matching posture allocation of obese individuals to that of lean may significantly increase EE, it is of fundamental importance to comprehensively establish whether or not the energy cost of standing posture maintenance is altered in the obese state. Indeed, body geometry, and more specifically the distribution of adipose mass, has been shown to influence postural stability (Corbeil et al., 2001; Gilleard and Smith, 2007; Blaszczyk et al., 2009; Singh et al., 2009; Cruz-Gomez et al., 2011; Villarrasa-Sapina et al., 2016). With increased abdominal obesity shown to increase postural sway, and presumably increased muscle work being required to maintain balance, one might hypothesize that the energy cost of postural maintenance may be elevated in individuals with abdominal obesity or certain body morphologies, although this remains to be tested.

## Energy cost vs. cardiovascular response

**Figure 5:**
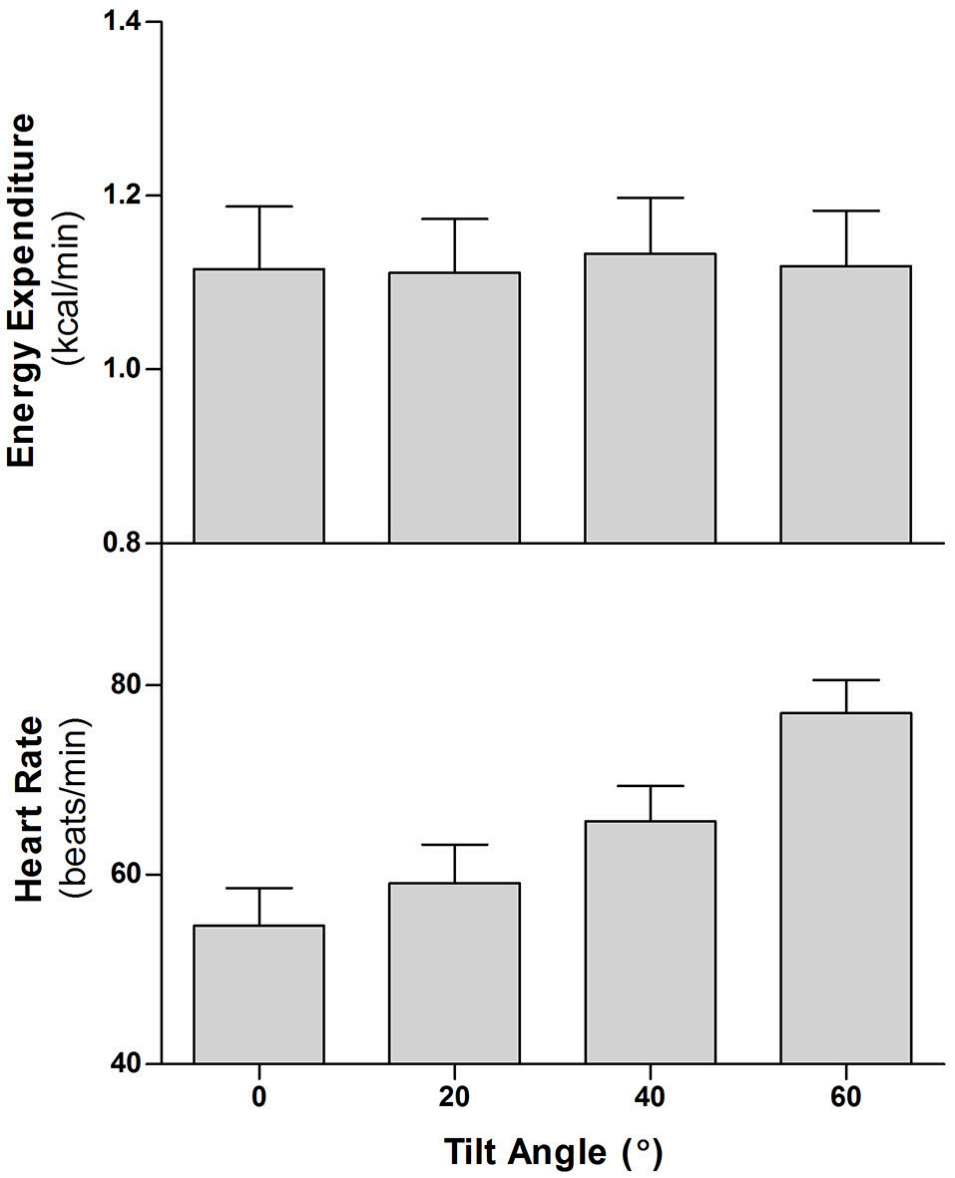
Energy expenditure (EE) and heart rate (HR) of 6 healthy men during graded, incremental head-up tilting on a clinical table. After a baseline measurement period of 40–45 min in the supine position, the subjects were passively tilted in increasing increments of 20 degrees (i.e., supine, 20°, 40°, 60°), remaining at each head-up tilt angle for 16 min. The motorized tilt table achieved each 20° of tilt within 4–5 s. Data are presented as Mean of last 4 min at each tilt angle ± SEM.

When considering the assessment of physical activity under free-living conditions, heart rate has traditionally been used as an objective, proxy measurement for EE. Indeed, while the recent advances in accelerometric devices are now allowing more accurate detection of body posture, commercially-available heart rate-based activity monitors are now widely used by the general public to monitor physical activity levels. However, it is important to note that although the relationship between these two variables is approximately linear during traditional, moderate-to-vigorous physical activity (Spurr et al., 1988), the same is not true of low-intensity physical activities (Ceesay et al., 1989). In order to maintain blood pressure during orthostasis, the autonomic nervous system works to increase both vasoconstriction in the extremities and heart rate. This increased heart rate persists across the standing period, and can occur in the absence of any obvious change in EE; as consistently observed in our recent studies where all individuals showed comparable increases in heart rate during steady-state standing (~15 beats per minute), despite responses in terms of EE ranging from little or no change compared to sitting to an increase of ~25% (Miles-Chan et al., 2013, 2017). Similarly, despite no detectable change in EE, we have also shown a significant difference in heart rate during sitting compared to supine ~7 beat per minute (Miles-Chan et al., 2014). Further, dissociation between the heart rate and EE response to altered body posture can be demonstrated in our preliminary study in healthy young men, performed using a clinical tilting table. With the body weight supported entirely by the tilting table, and thereby minimizing any muscular work required for posture transition and maintenance, we were able to observe a “dose-response” relationship between tilt angle (from supine to 60°) and heart rate, but no change in EE (Figure 5). Studies reporting values of EE estimated from heart rate in situations where postural allocation is not controlled (i.e., free-living conditions) should therefore be interpreted with considerable caution.

## Breaking the sedentary threshold

The energy cost of steady-state posture maintenance is relatively small (<35% above sitting). Bodily movements, e.g., displacement of the body (i.e., at least one step to be taken), are needed to increase EE beyond 1.5 times resting metabolic rate (Miles-Chan et al., 2017)—the level of EE commonly defined as the cut-off between sedentary and physical activities (Sedentary Behaviour Research Network, 2012). But there are two aspects of posture allocation that could potentially be exploited to increase EE, as described below.

### Energy cost of muscle activation (isometric contraction)

Maintaining posture, whether upright or seated, requires a certain degree of muscle tone and isometric contraction of stabilizing muscles. As the skeletal muscles involved in this stabilization and increased tonus are comprised of predominately oxidative fibers, increasing postural muscle activation could present not only an opportunity to increase EE, but also to increase the relative rate of fat oxidation. However, despite daily life activities consisting of a large amount of low-level isometric contraction, compared to dynamic exercise, its energy cost has been much less studied and quantified (Dulloo et al., 2017).

So how might isometric contraction be amplified in order to maximize EE during postural maintenance? Perhaps the simplest answer would be to alter posture allocation, so as to replace time spent in one posture with that of a potentially higher energetic cost (i.e., replace sitting time with standing time). However, this alone may not be sufficient to noticeably increase EE. Indeed, in addition to demonstrating that the majority of individuals (>75%) are able to maintain a standing posture at a similar level of EE to sitting (Miles-Chan et al., 2013, 2017), we have also shown that sitting in a comfortable chair, with the body weight well-supported, does not significantly increase EE above supine levels (<2% difference; Miles-Chan et al., 2014). In fact, based on these findings, replacing 2.5 h per day of lying or sitting by standing is in itself unlikely to increase daily EE by any more than 20 kcal (i.e., <1%); this is considerably less than the amount postulated by others (Levine et al., 2005; Johannsen et al., 2008). Similarly, Beers et al. (2008) have calculated that even sitting on a stability (exercise) ball—where the back is not supported—would still only result in an increase in sitting EE in the order of only around 0.07 kcal/min (~7%). This marginal increase in EE, combined with studies showing increased levels of discomfort when sitting on such a ball compared to a traditional office chair (Gregory et al., 2006; McGill et al., 2006; Kingma and van Dieen, 2009), suggest that the use of such sitting balls does not present an effective obesity prevention/treatment strategy.

However, several other methods of enhancing muscle activation during postural maintenance have demonstrated the ability to appreciably increase EE. For example: (i) whole body vibration during standing has been shown to increase expenditure by ~30% compared to standing without vibration (Fares et al., 2016); and (ii) Maffiuletti et al. (2012) have shown that standing in unstable shoes modestly increase EE (by ~5% on average) in patients with obesity as compared to conventional shoes, with increases in postural sway and electromyographic activity of the leg and foot muscle also having been demonstrated when using such shoes (Landry et al., 2010). It is perhaps worth noting that some of the large discrepancy in energetic response to these two methods of enhancing muscle activation may lie in the timescale of the muscle contraction itself—with studies in isolated muscle suggesting that a series of brief contractions may be more energetically costly than a single muscle contraction of a longer duration (Chasiotis et al., 1987; Bergstrom and Hultman, 1988; Hogan et al., 1998); the former also resulting in a larger increase in glycolysis and greater fatigue (Spriet et al., 1988; Hogan et al., 1998).

### Energy cost of postural transitioning and low-level physical activities

Whilst, the energy cost of maintaining posture may be marginal, the energy cost of transitioning between postures (in particular, from sitting to standing) is receiving much attention as a potential interventional target. The reasoning for this interest is two-fold: Firstly, breaking sitting time has been shown to decrease metabolic risk independently of moderate-to-vigorous physical activity (Honda et al., 2016), with length of sitting bouts positively correlated with waist circumference and obesity prevalence (Healy et al., 2008; Gupta et al., 2016), and frequent interruptions to sitting time improving postprandial glucose metabolism (Bergouignan et al., 2016), triglyceride levels, waist circumference and BMI (Hamilton et al., 2008; Healy et al., 2008). Secondly, the energy cost of postural transitioning is much higher than that of postural maintenance—with a sit-to-stand transition increasing EE ~35% above sitting metabolic rate (Judice et al., 2016), and showing a positive linear relationship with transition frequency (Hatamoto et al., 2016). Furthermore, the latter study (Hatamoto et al., 2016) demonstrated a four-fold increase in metabolic rate above resting during the performance of sit-to-stand transitions at a rate of 15 per minute, with the exercise still perceived as “light” by the participants. Importantly, while considerable inter-individual variability can be observed in the slope of this transition frequency vs. energy cost relationship, the cost is strongly correlated with body weight, thereby indicating that increasing postural transitioning may be of particular benefit to individuals who are overweight or obese (Hatamoto et al., 2016).

As mentioned earlier, in order to consistently increase EE beyond the sedentary threshold of 1.5 times resting metabolic rate (i.e., 1.5 METs), bodily movement is required. However, the physical activity need only be of a very low-level to achieve such an increase; with our own study finding that intermittent body displacement (stepping) increases EE to 1.5–1.6 METs (Miles-Chan et al., 2017). The low-level activities that comprise a large component of daily-life (e.g., domestic and household activities like carrying shopping, ironing, washing dishes, etc.) therefore present an ideal opportunity to elevate EE sufficiently to impact body weight management. The energetic cost of these activities was historically well-characterized in the context of estimating energy requirements (Passmore et al., 1952; Passmore and Durnin, 1955). Although, due to the myriad of technological advances made over recent decades, designed to make household activities quicker and easier, these early estimations are now largely redundant. There is hence a need to revisit such domestic activities in order to determine their contemporary energy cost. Recent investigations have shown that despite improved technologies, routine household activities easily reach energetic costs sufficient to be classified as low-intensity (>1.5 METs) to moderate-intensity (>3 METs; Gunn et al., 2002; Withers et al., 2006; Goh et al., 2016). To what extent the energy cost of these low-level physical activities of everyday life would differ if undertaken while standing compared to sitting (or vice versa) remains to be investigated. However, difficulties arise when comparing between population and study groups owing to a lack of standardized tests to assess the energy cost of low-level physical activity. Furthermore, there is a need to explore human variability in this cost, which may have important implications for the efficacy of the use of low-level physical activity for body weight management. With the majority of daily-life activities consisting of both isometric and dynamic activity (Dulloo et al., 2017), we have recently developed and validated two such standardized methodologies; one involving an isometric leg press protocol of low-intensity (Sarafian et al., 2013), and the other a low-intensity cycle ergometer protocol (Fares et al., 2017). These standardized approaches are applicable to a vast range of population groups (i.e., healthy, elderly, or diseased populations) and pave the way for a more comprehensive examination of inter-individual variability in both our susceptibility to obesity and the efficacy of body weight maintenance strategies.

## Concluding remarks

Whilst altering posture represents a simple target for body weight management, the gains in EE achieved by changing postural allocation *per-se* are unlikely to be of significant importance. However, increases in postural transitioning, either alone, or in combination with low-level physical activities presents a much more efficacious method; with the relatively minor increases in EE easily accumulated over the course of our daily activities. Whether, breaking the sedentary threshold will lead to compensatory increases in energy intake (or not) remains to be investigated. However, it should be emphasized that not only are these types of movements both attainable and sustainable by the majority of the general population, but such modest increases in physical activity may lead to a better coupling of energy intake to energy expenditure, and hence facilitate the achievement of energy balance—as suggested by the J-shaped curves of Mayer et al. (1956) and more recently revisited by Blundell et al. (2015) and Hopkins and Blundell (2016). Therefore, with suggestions that an energy imbalance of 100–200 kcal/day (i.e., <10% of average daily energy expenditure) may be sufficient to address the obesity crisis at the broad population level (Butte and Ellis, 2003; Hill et al., 2003), the role of posture allocations coupled with inter-individual variability in our metabolic response to low-level physical activities deserve considerable research attention.

## Author contributions

All authors listed, have made substantial, direct, and intellectual contribution to the work, and approved it for publication.

### Conflict of interest statement

The authors declare that the research was conducted in the absence of any commercial or financial relationships that could be construed as a potential conflict of interest.
